# Strategies to Counteract Oxidative Stress and Inflammation in Chronic-Degenerative Diseases 2.0

**DOI:** 10.3390/ijms25095026

**Published:** 2024-05-04

**Authors:** Cecilia Prata, Cristina Angeloni, Tullia Maraldi

**Affiliations:** 1 Department of Pharmacy and Biotechnology, Alma Mater Studiorum—University of Bologna, Via Irnerio 48, 40126 Bologna, Italy; 2 Department for Life Quality Studies, Alma Mater Studiorum—University of Bologna, Corso d’Augusto, 237, 47921 Rimini, Italy; 3 Department of Biomedical, Metabolic and Neural Sciences, University of Modena and Reggio Emilia, Via del Pozzo 71, 41125 Modena, Italy; tullia.maraldi@unimore.it

Oxidative stress and inflammation are recognized as pivotal contributors and common features of several chronic degenerative diseases, including cancer, metabolic syndrome, type 2 diabetes, cardiovascular diseases and neurodegenerative disorders, affecting a high percentage of the population [1,2]. Oxidative stress is characterized by an imbalance between the production of reactive oxygen species (ROS) and the body’s ability to detoxify them or repair the resulting damage [3]; inflammation is a natural and necessary biological response to protect the organism from injury, infection or exposure to harmful *stimuli.* Whereas acute inflammation is a beneficial short-term and localized response, chronic inflammation can be long-lasting and may contribute to various diseases and health issues [4]. Moreover, it has been observed that oxidative stress and chronic inflammation are involved in a vicious cycle where they mutually exacerbate each other [5,6].

Considering the increase in life expectancy and the fact that, currently, there are no effective cures for many chronic degenerative diseases, the possibility of identifying new tools to counteract these pathologies, preserving the structure and function of organs and tissues, is of paramount importance.

This second edition of the Special Issue explores multifaceted strategies aimed at counteracting oxidative stress and inflammation to mitigate their adverse health consequences. Thanks to the contribution of five original articles and three reviews, this Special Issue sheds light on new aspects in the fight against chronic degenerative diseases through the modulation of inflammation and oxidative stress by means of lifestyle modifications and therapeutic interventions. This Special Issue highlights emerging research in the field and identifies areas for future investigation, aiming at contributing to the advancement of preventive and therapeutic approaches to counteract chronic degenerative diseases.

In particular, four research articles of this Special Issue focus on the antioxidant and anti-inflammatory mechanisms of different natural or synthetic molecules: (1) Cardamonin, a chalconoid isolated from several traditional medicinal plants (*Alpinia katsumadai*, *Alpinia conchigera* and *Alpinia gagnepainii*), has recently been studied for its anticancer properties [7] and is here analyzed for its biological activities in LPS-activated BV-2 microglial cells. Microglia cells have to be included in neurodegeneration experimental models as they are involved in the neuroinflammation process [8,9]. (2) Astaxanthin, a red orange xanthophyll carotenoid which can be inserted into the cell membranes [10], is able to modulate biological functions related to lipid peroxidation [11]. Therefore, the paper delves into the role of natural astaxanthin in counteracting ferroptosis, a recently discovered process of cell death caused by excessive iron-dependent lipid peroxidation [12]. Ferroptosis is closely related to the pathophysiological processes of many oxidative-stress-related diseases [13], some of which are the topic of the other two research articles of the Special Issue: Parkinson disease [14] and renal fibrosis [15]. (3) Epoxidiol, an active metabolite of the antiparkinsonian monoterpenoid (1R,2R,6S)-3-methyl-6-(prop-1-en-2-yl)cyclohex-3-en-1,2-diol was analyzed in a rotenone-induced neurotoxicity model using in vitro, in vivo and ex vivo approaches, confirming the role of monoterpenoid in the improvement of Parkinson markers [16]. (4) MHY2013 is an agonist of Peroxisome Proliferator-Activated Receptors (PPARs). PPARs play an essential role in the regulation of various physiological processes, including lipid and energy metabolism. Here, the article explores renal fibrosis, the final pathological process of chronic kidney disease, which affects more than 10% of the world population and for which treatment options are limited, as described in detail in a recent review [17].

The above-mentioned research articles are based on antioxidants, describing their source and mechanism of action, their bioavailability and efficacy, and exploring their potential therapeutic applications in regulating oxidative stress and inflammation. It is well known that antioxidants play a pivotal role in neutralizing ROS by means of different mechanisms of action, providing protection from oxidative and inflammatory damage. Authors demonstrate in vitro and in vivo the importance of a balanced antioxidant defense system for maintaining cellular redox balance and preserving overall health.

This Special Issue also focuses on two different chronic degenerative diseases affecting the skeletal system, namely osteosarcoma and intervertebral disc degeneration. Here, there is a particular focus on osteosarcoma cell differentiation: beside the analysis of metabolic changes associated with mitochondrial morphology that take place during this energy-demanding process [18], the article sheds light on biomineralization. These important processes deserve attention in order to find new promising therapeutic strategies to counteract osteosarcoma, the primary malignant bone cancer affecting children and young adults [19]. A different, though still urgent, public health problem is intervertebral disc degeneration, experienced by more than 85% of people over 35 years of age [20]; the main manifestation of intervertebral disc degeneration is acute and chronic back pain caused by a combination of structural and mechanical deformities, as well as an increase in the activity of inflammatory mediators, including cytokines [21]. The most unfavorable factor, in terms of the rate of progression of intervertebral disc degeneration and the formation of vertebrogenic pain syndrome, is a high-producing cytokine imbalance. Classical therapeutic strategies for correcting cytokine imbalance in intervertebral disc degeneration do not give the expected response in more than half of all cases. As such, the aim of the review is to update knowledge about the molecular mechanisms of the imbalance between pro-inflammatory and anti-inflammatory cytokines, which are considered a new key to finding more effective drugs for the treatment of acute and chronic inflammation in patients with intervertebral disc degeneration.

In this Special Issue, the potential effects of lifestyle modifications on oxidative stress and inflammation are explored in relation to diabetes. Undoubtedly, oxidative stress and inflammation play a major role in the pathogenesis of this disease, and the low antioxidant capacity of pancreatic β-cells results in pancreatic cell failure in both type 1 and 2 diabetes [22]. Oxidative stress leads to reduced insulin production, impaired insulin secretion, β-cell dysfunction and its apoptosis, reduced expression of the GLUT-4 receptor and impairment of the insulin signal transduction pathway. These processes, as well as persistent, low-grade inflammation, lead to insulin resistance and type 2 diabetes [23]. The review discusses why an important strategy to control, in particular but not only, type 2 diabetes is to lead a healthy lifestyle based on, among other things, regular physical activity, giving up smoking, following a balanced diet containing ingredients with antioxidant potential, including vegetables and fruits, and these correct behaviors are strongly suggested in the presence or absence of treatment with hypoglycemic pharmacotherapy.

As a sort of conclusion, a comprehensive review delves into the intricate pathway underlying oxidative stress, including the generation of ROS, cellular targets of oxidative damage, and the ensuing disruption of cellular homeostasis. By elucidating these cascades, one of the three reviews in this Special Issue aims to provide a comprehensive overview of the molecular mechanisms involved in counteracting oxidative stress, chronic inflammation and consequently aging, together with chronic degenerative pathologies.

The vastness and complexity of the topic of the two editions of this Special Issue highlight the importance of interdisciplinary collaboration and ongoing research endeavors in advancing our understanding and management of oxidative stress and inflammation-associated pathologies and consequently counteracting them and promoting people’s health span. By harnessing our collective knowledge and resources, we can pave the way for innovative strategies to enhance overall well-being.

The third edition of this Special Issue, which is currently open to new submissions, will provide additional scientific evidence in order to contribute towards new understandings of chronic degenerative diseases and new preventive or co-treatment strategies. 
